# Associations of Food-Chewing Discomfort with Health Behaviors and Cognitive and Physical Health Using Pooled Data from the Korean Health Panel (2010–2013)

**DOI:** 10.3390/nu12072105

**Published:** 2020-07-16

**Authors:** Sun Mi Shin

**Affiliations:** Department of Nursing, Joongbu University, Chubu-myeon, Geumsan-gun, Chungcheongnam-do 32713, Korea; healthteam@joongbu.ac.kr; Tel.: +82-10-9288-5177

**Keywords:** food-chewing discomfort, health behavior, smoking, drinking, physical activity, memory decline, decision-making, chronic disease

## Abstract

Using 4 years of pooled data from the Korean Health Panel (2010–2013), the prevalence of food-chewing discomfort in adults over the age of 19 was investigated and the cross-sectional relationship between food-chewing discomfort and health behaviors and cognitive and physical health was identified. The prevalence of food-chewing discomfort was 31%: young adults (<40 years), 17.9%; middle-aged adults (40–64 years), 28.9%; and older adults (≥65 years), 57.1% (*p* < 0.0001). When food-chewing discomfort was sometimes, often, or always rather than never, odds ratios (ORs) were analyzed after controlling for sociodemographic characteristics. Significant OR results of target variables were smoking (OR 1.15, 1.37, 1.50), drinking (1.08, 0.87, 0.73), problem drinking (1.87, 1.67, 1.34), abstinence from drinking (1.23, 1.34, 1.42), nonphysical activity (OR 0.87 only significant, 0.94 nonsignificant, 1.10 nonsignificant), memory decline (2.07, 2.56, 3.31), decision-making difficulty (1.76, 2.78, 4.37), limitation of daily life due to illness (2.29, 3.60, 3.92), and the presence of a chronic disease (1.28, 1.62, 1.73), respectively. In conclusion, there were associations of food-chewing discomfort with increased smoking and decreased alcohol consumption, with increased difficulty in decision-making and memory decline, limitations in daily life due to disease, and the presence of chronic diseases. Therefore, it is necessary to investigate the causal relationship between chewing and health behaviors and cognitive and physical health through longitudinal studies.

## 1. Introduction

All living organisms maintain dynamic homeostasis from internal and external stress [1,2]. Homeostasis begins with feeding, and feeding begins with chewing food. Through chewing, the contents of the oral cavity are broken and ground, and through this process, food is divided into sizes effective for digestion, absorption, and swallowing [1,3]. The human body can then effectively consume nutrients. Food chewing is related to nutrition and plays a central role in human health. In addition, chewing ability is related not only to dietary intake for nutrients, but also to oral-health status, chronic disease, and quality of life [4,5]. Therefore, it is necessary to recognize if chewing discomfort is associated with health behavior and cognitive and physical health.

Discomfort while chewing food is caused by complete or partial loss of teeth, pain caused by stomatitis or periodontitis, misalignment of the upper and lower teeth, loose teeth, orthodontic appliances or dentures, and lack of teeth [4,5]. The neuroendocrine response to stress is achieved through the activation of the hypothalamic-pituitary-adrenal (HPA) axis [2,6]. Activation of the HPA axis is a source of stress, which is suppressed through chewing. Therefore, the ability to chew plays an important role in physical and mental social health. In experiments, when the HPA was activated, the circulation of glucocorticoids in the body increased, leading to a decrease in hippocampus-dependent cognitive function and causing various chronic diseases, such as hypertension, cardiovascular disease, and osteoporosis [3,5,7,8,9,10,11].

In previous studies, eating through chewing has been evaluated in several ways. Here, survey information about food-chewing discomfort, subjectively revealed by each individual, was objectively reliable and valid information [12,13]. Nevertheless, the problem of chewing discomfort was mainly targeted at older adults, and the relationship between senile dementia and fatal chronic diseases was explored. However, the direct factor of chewing discomfort is caused by physical factors, such as tooth and oral pain, dentition, chewing power, and aging [14,15]. Therefore, chewing discomfort may occur in the population of any age group, and it is important in terms of oral-health policy to determine the prevalence of food-chewing discomfort for the whole population. Identifying the relationship between food-chewing discomfort and health outcomes would also be meaningful in terms of nutritional epidemiology.

The hypothesis of this study was first that there would be inconvenience in chewing food even in young and middle-aged people. Second, discomfort in chewing food was set to be related to health behaviors of smoking, drinking, physical activity, and cognitive and physical health. Therefore, the purpose of this study was to determine the prevalence of food-chewing discomfort in adult populations over the age of 19 and to assess the relationship between food-chewing discomfort and health behaviors and cognitive and physical health.

## 2. Materials and Methods

### 2.1. Study Design

This research is a cross-sectional study to know the relationship between food-chewing discomfort and health behaviors and cognitive and physical health in an adult population using 4 years of pooled Korean Health Panel (KHP) data, from 2010 to 2013.

### 2.2. Data

This study used pooled data from the KHP from 2010 to 2013. Since 2008, KHP data has been researched every year to understand the health level of Koreans and develop needed medical policies. In order to maintain the population representative, KHP data were based on the extraction frame of 90% of the total population survey. The sample was selected by the two-stage probability-proportional stratified sampling method, which first extracted the provinces by the systematic sampling method and then the sample households. The sample size was approximately 8000 households nationwide with household members belonging to the household, so that a certain level of households could be maintained in consideration of the dropout rate. Although it is a principle that the same subjects are continuously surveyed every year, it is an open panel that imports new households in the same way when detached households occur due to death, rejection, etc.

The KHP survey on sociodemographic characteristics, the presence of disease, and medical utilization began in 2008. In addition, since 2009, further surveys on health behaviors and quality of life have been initiated for adults over the age of 19 and then merged. Survey variables, such as new entries and termination, have been changed according to research years and needs, also providing population-based weights to complement unequal selection probabilities and nonresponses and to match population and sample distributions.

For KHP surveys every year, experienced investigators visit homes and conduct computer-based interviews. The Korea Institute for Health and Social Affairs (KIHASA), a nationally accredited data-management agency, reviews data collected by investigators; then, through KHP conference, it improves the data again and publicly discloses it to researchers about 3 years later. KHP data used in this study were delivered by KIHASA through an official route and approved by the institutional review committee (KIHASA 2016-01).

### 2.3. Study Subjects

The period of the survey of major variables used in this study spanned only 4 years, from 2010 to 2013. Therefore, the KHP dataset for this study was 4 years of pooled panel data, and the data held 49,120 subjects. For the homogeneity of the study subjects, the final 44,072 (89.7%) were selected for analysis in this study after excluding 2412 (4.9%) medical-aid and 2636 (5.4%) disabled subjects, who belonged to extreme poverty.

### 2.4. Study Variables and Term Definitions

Main variables in this study were food-chewing discomfort, health behaviors, and cognitive and physical health. Discomfort in chewing food used response variables “never”, “sometimes”, “often”, and “always” to the question “have you ever had difficulty in chewing food due to problems in your mouth, such as teeth, in the past year?” In this study, the prevalence of food-chewing discomfort was identified by classifying it as nonexistent (never) and existent (sometimes, often, or always).

The variables for sociodemographic characteristics were gender (male, female), survey year (2010, 2011, 2012, 2013), age group (young adults (<40 years), middle-aged adults (40–64 years), older adults (≥65 years), illiteracy (yes, no), marital status (unmarried, married, bereavement, and divorce), and economic activity (yes, no). Between them, marital status was reclassified as having a spouse (married) or not (unmarried, bereavement, and divorce), and we used it as a covariate.

In this study, smoking, drinking, abstinence from drinking, problem drinking, and physical activity were used to identify health behavior [16]. In the last year, the three variables for alcohol consumption were drinking (nondrinking and drinking), abstinence (non-abstinence and abstinence), and problematic drinking (nonproblematic drinking and problematic drinking). A problem drinker is a person who has had trouble in their daily life due to drinking [17]. Nondrinking included lifelong nondrinkers and one-year nondrinkers, and non-abstinence included drinkers and lifelong nondrinkers. In addition, nonproblematic drinking included drinkers, nondrinkers, and abstainers.

In this study, physical activity was measured using KHP raw materials, surveyed according to the International Physical Activity Questionnaire (IPAQ). The questions given in the questionnaire to find vigorous physical activity and its amount were as follows: “how many days have you spent more than 10 min of strenuous physical activity in the past week that has caused much shortness of breath and greatly increased heart rate?” and “how many minutes did you usually do on one day?”. To find moderate physical activity and its amount, the questions were: “how many days have you spent more than 10 min of moderate physical activity in the past week that has caused moderate shortness of breath and slightly increased heart rate?” and “how many minutes did you usually do on one day?”. To find low physical activity and its amount, questions were: “how many days did you walk more than 10 min a day in the past week?” and “how many minutes did you usually walk on one day?”

The amount and intensity of physical activity responded by the subjects were calculated as metabolic-equivalent tasks (MET, mins) [18,19]. The following values were used for analysis of these study data: walking MET-min/wk = 3.3 × walking mins × walking days; moderate MET-min/wk = 4.0 × moderate-activity mins × moderate days; vigorous MET-min/wk = 8.0 × vigorous mins × vigorous days.

These were classified into three categories of physical activity (low, moderate, and high), according to the IPAQ scoring system. Low/inactive was the lowest level of physical activity. Subjects who did not meet the criteria for the moderate or high categories were considered low/inactive. ‘Moderate’ related to any one of the three following criteria: 3 or more days of vigorous activity of at least 20 min per day; 5 or more days of moderate activity or walking of at least 30 min per day; or 5 or more days of any combination of walking, moderate, or vigorous activities, achieving a minimum of at least 600 MET-min/week. ‘High’ related to either one of the 2 following criteria: vigorous activity on at least 3 days and accumulating at least 1500 MET-min/wk or 7 or more days of any combination of walking, moderate, or vigorous activities, achieving a minimum of at least 3000 MET-min/wk.

In addition, in this study, in order to identify odds ratios (ORs) of nonphysical activity according to food-chewing discomfort, low/inactive physical activity was defined as the nonphysical-activity group and moderate and high physical activity was defined as the physical-activity group.

Decision making and memory can be used as indicators of cognitive health [20,21]. As variables for cognitive health, this study analyzed two variables, memory decline (yes, no), and difficulty in decision-making (yes, no). Memory decline is when subjects have had trouble in their daily lives due to confusion or memory loss, and decision-making difficulty is when they had difficulty making decisions to the extent that it interferes with their daily lives.

### 2.5. Analysis

The prevalence of food-chewing discomfort and sociodemographic characteristics, health behaviors, and cognitive and physical health of the adult population were calculated via a chi-squared test. At this time, the representativeness of the sample was confirmed through analysis using population-based weights.

In this study, associations of food-chewing discomfort with health behavior and cognitive and physical health were calculated by the odds ratios (ORs) through multiple logistic-regression models. An odds ratio compares the relative likelihood of an event (outcome) occurring between independent groups [22]. This time, sociodemographic variables were used as covariates. All analyses were conducted using SAS 9.4 (SAS Institute, Cary, NC, USA). Each statistical result was interpreted as significant when the *p*-value was less than the significance level of 0.05.

## 3. Results

### 3.1. Sociodemographic Characteristics of Subjects

Among 44,072 subjects, 46.3% were male and 53.7% were female. In sociodemographic characteristics divided by males and females, the proportion of subjects who reported that food chewing was always uncomfortable was 18.5% and 21.4% (*p* < 0.001) for those over 65 years old, 0.2% and 2.5% for illiteracy (*p* < 0.0001), 74.5% and 68.2% for marriage (*p* < 0.0001), 76.9% and 50.3% for economic activity (*p* < 0.0001), and 3.9% and 4% (Table 1).

### 3.2. Prevalence of Food-Chewing Discomfort by Sociodemographic Characteristics

The prevalence of food-chewing discomfort was 31% in the adult population aged 19 years and older in Korea. It was lowest to highest in subjects for this order: young adults (<40 years), middle-aged adults (40–64 years), and older adults (≥65 years) (17.9% vs. 28.9% vs. 57.1%, respectively, *p* < 0.0001); and was lowest to highest for subjects in this order: unmarried, married, and bereavement or divorced (18.6% vs. 31.4% vs. 52.0%, respectively, *p* < 0.0001). In addition, it was higher in illiterate than literate subjects (64.0% vs. 30.6%, respectively, *p* < 0.0001), and higher in noneconomic than in economic activity subjects (33.5% vs. 29.5%, respectively, *p* < 0.0001) (Table 2).

### 3.3. Prevalence of Food-Chewing Discomfort by Health Behaviors

The prevalence of food-chewing discomfort was higher in smokers than in nonsmokers (32.1% vs. 30.7%, respectively, *p* < 0.01); lower in drinkers than nondrinkers for the past 1 year (28.6% vs. 37.1%, respectively, *p* < 0.0001); higher in problem drinkers than nonproblem drinkers (37.0% vs. 30.3%, respectively, *p* < 0.0001); much higher in abstainers from drinking than nonabstainers (41.4% vs. 30.4%, respectively, *p* < 0.0001); and slightly higher in those performing moderate and high physical activity than those doing low physical activity (31.9% vs. 31.7% vs. 30.1%, respectively, *p* < 0.0003; Table 3).

### 3.4. Prevalence of Food-Chewing Discomfort by Cognitive and Physical Health

The prevalence of food-chewing discomfort was much higher when memory decline was “yes” rather than “no” (60.3% vs. 29.9%, *p* < 0.0001), when decision-making difficulty was “yes” rather than “no” (66.2% vs. 30.6%, *p* < 0.0001), when daily-life problems due to illness were “yes” rather than “no” (69.7% vs. 29.7%. *p* < 0.0001), and when presence of chronic disease was “yes” rather than “no” (38.2% vs. 21.2%, *p* < 0.0001) (Table 4).

### 3.5. Odds Ratios of Health Behavior by Degree of Food-Chewing Discomfort

The ORs of each health behavior were determined according to the degree of food-chewing discomfort. When food-chewing discomfort was “sometimes”, “often”, and “always”, rather than “never”, the OR of smoking was 1.15 (95% CI 1.07–1.24), 1.37 (95% CI 1.25–1.50), and 1.50 (95% CI 1.30–1.73); the OR of drinking was 1.08 (95% CI 1.01–1.15), 0.87 (95% CI 0.81–0.93), and 0.73 (95% CI 0.65–0.81); the OR of problem drinking was 1.87 (95% CI 1.73–2.02), 1.67 (95% CI 1.50–1.85), and 1.34 (95% CI 1.12–1.60); the OR of abstinence from drinking 1.23 (95% CI 1.11–1.37), 1.34 (95% CI 1.18–1.51), and 1.42 (95% CI 1.20–1.69), respectively. When chewing discomfort was “sometimes” rather than “never”, the OR of nonphysical activity only had a significance of 0.87 (95% CI 0.83–0.92; Table 5).

### 3.6. Odds Ratios of Cognitive and Physical health by Degree of Food-Chewing Discomfort

In order to investigate cognitive health by the degree of food-chewing discomfort, ORs of memory decline and decision-making difficulty were identified. When discomfort in chewing food was “sometimes”, “often”, and “always”, rather than “never”, the OR of memory decline was 2.07 (95% CI 1.81–2.36), 2.56 (95% CI 2.22–2.95), and 3.31 (95% CI 2.77–3.96); and the OR of decision-making difficulty was 1.76 (95% CI 1.37–2.27), 2.78 (95% CI 2.16–3.57), and 4.37 (95% CI 3.29–5.82), respectively (Table 6).

In order to know physical health by the degree of food-chewing discomfort, ORs of limitations of daily life due to illness and the presence of chronic disease were identified. When discomfort in chewing food was “sometimes”, “often”, and “always”, rather than “never”, the OR of limitations of daily life due to illness was 2.29 (95% CI 1.98–2.64), 3.60 (95% CI 3.11–4.16), and 3.92 (95% CI 3.26–4.72); ORs of the presence of chronic disease were 1.28 (95% CI 1.21–1.36), 1.62 (95% CI 1.50–1.76), and 1.73 (95% CI 1.51–1.98), respectively (Table 6).

## 4. Discussion

Continued dental care is necessary even at a young age. The need to chew food thoroughly from an early age is important for early health education, emphasized in any country. In Korea, there is a traditional saying: habits at the age of three last a lifetime. This proverb is emphasized and practiced in all areas of health. Chewing ability may be worse due to poor dental or oral health, and it is known to have a great influence on health [4,23]. However, interest in food chewing has mostly been targeted at older adult populations. Food-chewing discomfort caused by problems with teeth or oral cavities is a health problem that can occur regardless of age. Therefore, exploring the relationship between health and food-chewing discomfort in adults is the basis for health promotion. In particular, studies on food-chewing discomfort and health behavior are very rare, and associations of food chewing with cognition and physical health have mainly been published based on elderly populations.

Here, prevalence of food-chewing discomfort was 31% in the adult population, aged 19 years and older. Differences in gender prevalence were not significant, with males and female sat 31.4% and 30.7%, respectively. The prevalence of chewing difficulty in adults over 45 years was 20% in Florida and 30% in Taiwan [13,24]. However, the prevalence of chewing ability is difficult to compare and depends on the diagnostic criteria for each study. On the other hand, as age increases, it is more likely to experience difficulty in chewing [24,25,26]. In this study, the prevalence of chewing discomfort in older adults aged 65 and over was 57.1%, which was much higher than the 28.9% in the middle-aged population, aged 40 to 64, and the 17.9% in the young-adult population, under the age of 40. However, the prevalence of chewing discomfort in young adults under the age of 40 was high enough that it could not be ignored in terms of nutritional epidemiology, considering eating and health.

In this study, the prevalence of food-chewing discomfort was higher in low socioeconomic conditions. The reason was that the prevalence of chewing discomfort was higher in illiterate people than those who were literate (64.0% and 30.6%) and in married, separated, or divorced people than unmarried people (31.4%, 52.0%, and 18.6%, respectively). It was also higher in those not performing economic activities than those who did (33.5% and 29.5%). In previous studies, when economic level was low, barriers to the use of medical services for dental visits were high [27,28,29]. In childhood, environments causing vulnerability and low health levels were related to future dental-health levels, and widows had a higher rate of chewing discomfort [30,31]. In addition, dental health was worse when education was low [32].

With regard to the association of food-chewing discomfort with smoking, even after controlling for sociodemographic characteristics, the ORs of smoking were “sometimes” 1.15 (95% CI 1.07–1.24), “often” 1.37 (95% CI 1.25–1.50), and “always” 1.50 (95% CI 1.30–1.73). In previous studies, tobacco consumption also affected periodontal and dental injuries and could worsen oral hygiene [32,33]. However, this study strongly suggests a longitudinal study, because this was a cross-sectional study, in which the causal relationship is unknown.

Drinking, especially problem drinking, is a behavior that is dangerous for health, which can adversely affect not only an individual’s health, but also their family, neighbors, work, and social activities. As a result of this study, drinking behavior changed positively when there was discomfort in chewing. Even after controlling for confounding variables, such as age, gender, literacy, spouse, and economic activity, the ORs of drinking were “sometimes” 1.08 (95% CI 1.01–1.15), “often” 0.87 (95% CI 0.81–0.93), and “always” 0.73 (95% CI 0.65–0.81). In addition, the ORs of problem drinking were also increased to 1.87 (95% CI 1.73–2.02), 1.67 (95% CI 1.50–1.85) and 1.34 (95% CI 1.12–1.60), respectively. ORs of abstinence from drinking also increased to 1.23 (95% CI 1.11–1.37), 1.34 (95% CI 1.18–1.51), and 1.42 (95% CI 1.20–1.69), respectively. Therefore, there was a correction of drinking behavior in subjects who perceived food-chewing discomfort as a health threat.

As a result of this study, it can be see that when there is discomfort in chewing food, physical activity beneficial to health should be improved. The reason for this is that the OR of “nonphysical activity” decreased to 0.87 (95% CI 0.83–0.92) when food-chewing discomfort was “sometimes” rather than “never”. Therefore, it is necessary to develop a health-promotion program for adult populations who “sometimes” have discomfort in chewing and who are aware of the health threats. On the other hand, in a previous study, the ability to chew was a factor that could independently predict physical fitness, such as strength of leg muscles [34]. Therefore, when chewing discomfort is “always”, physical activity using muscle strength can be predicted to be relatively less. However, in this study, the relationship between subjects “always” having chewing discomfort and doing “nonphysical activity” was not statistically significant.

There were many cognitive impairments in people with loss of teeth or poor chewing ability [35,36,37]. In this study, to investigate the association of food-chewing discomfort with cognitive health, variables of memory decline and decision-making difficulty were analyzed. After controlling for sociodemographic characteristics, the ORs of memory decline when chewing discomfort was “sometimes”, “often”, and “always”, rather than “never”, were 2.07 (95% CI 1.81–2.36), 2.56 (95% CI 2.22–2.95), and 3.31 (95% CI 2.77–3.96), respectively. In addition, ORs of decision-making difficulty increased to 1.76 (95% CI 1.37–2.27), 2.78 (95% CI 2.16–3.57), and 4.37 (95% CI 3.29–5.82), respectively.

On the basis of the mechanism of chewing and homeostasis [3,5,7,8,9,10,11], chewing ability may be associated with cognitive impairment and a chronic disease that requires long-term management. In this study, “limitation of daily life due to illness” and “presence of chronic disease” were analyzed. After controlling for sociodemographic characteristics when discomfort in chewing food was “sometimes”, “often”, and “always”, rather than “never”, the ORs of daily-life limitations due to illness were 2.29 (95% CI 1.98–2.64), 3.60 (95% CI 3.11–4.16), and 3.92 (95% CI 3.26–4.72), respectively. In addition, the ORs of the presence of a chronic disease increased to 1.28 (95% CI 1.21–1.36), 1.62 (95% CI 1.50–1.76), and 1.73 (95% CI 1.51–1.98), respectively. In previous studies, it was also associated with severe disease, such as chronic kidney disease or chronic obstructive pulmonary disease [38,39].

### Limitations

This study used pooled panel data, representing Koreans from 2010 to 2013, but designed a cross-sectional study due to the short period. As a result, in regards to food-chewing discomfort alongside health behaviors and outcomes, there is a limitation, due to the fact that we are not able to explain causal relationships. Therefore, it is necessary to study the causal relationship between food-chewing discomfort and health outcomes through longitudinal-study design.

In this study, the subjective variables of food-chewing discomfort were used to explore only some of the macroscopic topics of food intake and health. This was because KHP data used in this study did not include information such as the number or condition of teeth or the type and number of drugs taken. Therefore, in a future study, it is necessary to study the links of variables related to food-chewing discomfort, medical-examination data from hospitals, and drug-use information.

In addition, all variables used in this study are subjective information because they are based on the subject questionnaire. Therefore, there may be differences from clinical data based on actual measurements and examinations.

## 5. Conclusions

Food-chewing discomfort can be caused not only by aging, but also by physical problems, such as those in the teeth and the oral cavity. However, there are few studies on the relationship between food-chewing discomfort and health in middle-aged (40–64 years) and young (<40 years) adults.

Conclusively, the prevalence of food-chewing discomfort in middle-aged (40–64 years) and young (<40 years) adults was lower than in older adults (≥65 years), but it was not low in terms of nutritional epidemiology. In addition, the prevalence of food-chewing discomfort increased in smoking, decreased in drinking and problem drinking, and increased in abstinence. Only in those who sometimes felt chewing discomfort rather than never, nonphysical activity decrease. The prevalence of food-chewing discomfort also increased in memory decline, decision-making difficulty, and limitations of daily life due to illness and the presence of chronic disease.

## Figures and Tables

**Table 1:** Sociodemographic characteristics of study subjects, and sampling weights.

Classification		Subjects						Chi-Squared/*p*-Value	
		Total	(%)	Male	(%)	Female	(%)	Subjects	Population-Based Weights
All		44,072	(100.0)	20,414	(46.3)	23,658	(53.7)		
Survey year	2010	11,898	(27.0)	5515	(27.0)	6833	(27.0)	0.13/0.98	0.16/<0.98
	2011	11,359	(25.8)	5274	(25.8)	6085	(13.8)		
	2012	10,702	(24.3)	4954	(24.3)	5748	(24.3)		
	2013	10,113	(23.0)	4671	(22.9)	5442	(23.0)		
Age group	Youth (<40)	14,023	(31.8)	6422	(31.5)	7601	(32.1)	76.73/<0.0001	146.60/<0.0001
	Middle aged (<65)	21,223	(48.2)	10,222	(50.1)	11,001	(46.5)		
	older adults (≥65 years)	8826	(20.0)	3770	(18.5)	5056	(21.4)		
Illiteracy	Illiteracy	638	(1.5)	41	(0.2)	597	(2.5)	414.35/<0.0001	351.94/<0.0001
	Literacy	43,430	(98.6)	20,371	(99.8)	23,059	(97.5)		
Marital status	Unmarried	8317	(18.9)	4437	(21.7)	3880	(16.4)	1712.45/<0.0001	1501.63/<0.0001
	Married	31,355	(71.1)	15,214	(74.5)	16,141	(68.2)		
	Separated, divorced	4400	(10.0)	763	(3.7)	3637	(15.4)		
Economic activity	Yes	27,582	(62.6)	15,690	(76.9)	11,892	(50.3)	3309.39/<0.0001	4024.69/<0.0001
	No	16,490	(37.4)	4724	(23.1)	11,766	(49.7)		
Chewing discomfort	Never	30,396	(69.0)	14,001	(68.6)	16,395	(69.3)	20.22/<0.0001	55,283.11/<0.0001
	Sometimes	7364	(16.7)	3574	(17.5)	3790	(16.1)		
	Often	4558	(10.3)	2040	(10.0)	2518	(10.3)		
	Always	1754	(4.0)	799	(3.9)	955	(4.0)		

**Table 2:** Prevalence of food-chewing discomfort by sociodemographic characteristics.

Classification		Chi-Squared/*p*-Value						
		Total	Nonexistent	(%)	Existent	(%)	Subjects	Population-Based Weights
All		44,072	30,396	(69.0)	13,676	(31.0)		
Gender	Male	20,414	14,001	(68.6)	6413	(31.4)	2.62/0.1058	5.30/0.0213
	Female	23,658	16,395	(69.3)	7263	(30.7)		
Age group	Young adults (<40)	14,023	11,515	(82.1)	2508	(17.9)	3976.68/<0.0001	3723.84/<0.0001
	Middle aged (<65)	21,223	15,093	(71.1)	6130	(28.9)		
	Older adults (≥65 years)	8826	3788	(42.9)	5038	(57.1)		
Literacy	Literacy	43,430	30,164	(69.5)	13,266	(30.6)	327.83/<0.0001	321.42/<0.0001
	Illiteracy	638	230	(36.1)	408	(64.0)		
Marital status	Unmarried	8317	2113	(48.0)	1548	(18.6)	1503.18/<0.0001	1374.92/<0.0001
	Married	31,355	6769	(81.4)	9841	(31.4)		
	Separated, divorced	4400	21,514	(68.6)	2287	(52.0)		
Economic activity	Yes	27,582	19,437	(70.5)	8145	(29.5)	77.59/<0.0001	24.60/<0.0001
	No	16,490	10,959	(66.5)	5531	(33.5)		

**Table 3:** Prevalence of food-chewing discomfort by health behavior.

Classification		Chi-Squared/*p*-Value						
		Total	Nonexistent	(%)	Existent	(%)	Subjects	Population-Based Weights
All		44,072	30,396	(69.0)	13,676	(31.0)		
Smoking	No	34,480	23,881	(69.3)	10,599	(30.7)	6.35/0.0117	73,117.59/<0.0001
	Yes	9590	6513	(67.9)	3077	(32.1)		
Drinking for past one year	No ^1^	12,544	7889	(62.9)	4655	(37.1)	302.71/<0.0001	550,764.00/<0.0001
	Yes	31,528	22,507	(71.4)	9021	(28.6)		
Abstinence from drinking	No ^2^	41,430	28,847	(69.6)	12,583	(30.4)	140.38/<0.0001	331,448.00/<0.0001
	Yes	2642	1549	(58.6)	1093	(41.4)		
Problem drinking for past one year	No ^3^	39,318	27,403	(69.7)	11,915	(30.3)	89.98/<0.0001	504,246.00/<0.0001
	Yes	4754	2993	(63.0)	1761	(37.0)		
Physical activity in the past week	Low/inactive	20,626	14,420	(69.9)	6206	(30.1)	16.15/0.0003	1351.46/<0.0001
	Moderate	17,573	11,967	(68.1)	5606	(31.9)		
	High	5873	4009	(68.3)	1864	(31.7)		

Note: ^1^: nondrinkers, namely, lifelong nondrinkers and abstainers from drinking; ^2^: nonabstainers, namely, drinkers and nondrinkers; and ^3^: nonproblem drinkers, namely, drinkers, nondrinkers, and abstainers.

**Table 4:** Prevalence of food-chewing discomfort by cognitive and physical health.

Classification		Chi-Squared/*p*-Value						
		Total	Nonexistent	(%)	Existent	(%)	Subjects	Population-Based Weights
All		44,072	30,396	(69.0)	13,676	(31.0)		
Memory decline	No	42,463	29,757	(70.1)	12,706	(29.9)	667.81/<0.0001	595.90/<0.0001
	Yes	1609	639	(39.7)	970	(60.3)		
Decision-making difficulty	No	43,587	30,232	(69.4)	13,355	(30.6)	283.18/<0.0001	244.28/<0.0001
	Yes	485	164	(33.8)	321	(66.2)		
Daily-life limitations due to illness	No	42,590	29,947	(70.3)	12,643	(29.7)	1071.64/<0.0001	1049.92/<0.0001
	Yes	1482	449	(30.3)	1033	(69.7)		
Chronic disease	No	18,618	14,665	(78.8)	3953	(21.2)	1446.25/<0.0001	1200.62/<0.0001
	Yes	25,454	15,731	(61.8)	9723	(38.2)		

**Table 5:** Odds ratios of health behaviors by food-chewing discomfort through multiple logistic regression.

Classification	Reference		Smoking (*n* = 9589)	Drinking (*n* = 31,525)	Problem Drinking (*n* = 4754)	Abstinence from Drinking (*n* = 2642)	Nonphysical Activity ^1^ (*n* = 14,087)
Gender	Male	Female	34.43	3.01	4.69	1.12	0.68
			(31.34–37.82)	(2.86–3.16)	(4.35–5.06)	(1.03–1.21)	(0.65–0.70)
Age group	Young adults (<40)	Older adults (≥65)	1.95	4.16	3.92	0.52	1.1
			(1.77–2.14)	(3.88–4.45)	(3.45–4.46)	(0.46–0.58)	(1.01–1.20)
	Middle-aged adults (<65)		1.83	2.63	2.89	0.50	0.75
			(1.68–1.99)	(2.48–2.79)	(2.56–3.25)	(0.45–0.55)	(0.71–0.79)
Literacy	Illiteracy	Literacy	3.09	0.83	0.51	1.25	2.07
			(2.19–4.37)	(0.70–0.99)	(0.24–1.08)	(0.96–1.64)	(1.74–2.47)
Spouse	No	Yes	1.36	0.91	0.96	0.53	1.14
			(1.27–1.47)	(0.86–0.96)	(0.88–1.04)	(0.48–0.59)	(1.09–1.20)
Economic activity	Yes	No	1.76	1.62	1.57	0.57	1.03
			(1.64–1.89)	(1.55–1.71)	(1.44–1.70)	(0.52–0.62)	(0.99–1.08)
Food chewing discomfort	Sometimes	Never	1.15	1.08	1.87	1.23	0.87
			(1.07–1.24)	(1.01–1.15)	(1.73–2.02)	(1.11–1.37)	(0.83–0.92)
	Often		1.37	0.87	1.67	1.34	0.94
			(1.25–1.50)	(0.81–0.93)	(1.50–1.85)	(1.18–1.51)	(0.88–1.00)
	Always		1.50	0.73	1.34	1.42	1.10
			(1.30–1.73)	(0.65–0.81)	(1.12–1.60)	(1.20–1.69)	(0.99–1.20)

Note: 1: Nonphysical activity means low/inactive physical activity.

**Table 6:** Odds ratios of cognitive and physical health by food-chewing discomfort through multiple logistic regression.

Classification	Criteria		Cognitive Health		Physical Health	
			Memory Decline (*n* = 1609)	Decision-Making Difficulty (*n* = 484)	Limitation of Daily Life Due to Illness (*n* = 1480)	Presence of Chronic Disease (*n* = 25,451)
Gender	Female	Male	0.76	1.10	0.82	0.60
			(0.68–0.85)	(0.90–1.35)	(0.73–0.93)	(0.57–0.63)
Age group	Young adults (<40)	Older adults (≥65)	0.26	0.22	0.09	0.04
			(0.22–0.31)	(0.16–0.30)	(0.07–0.11)	(0.04–0.04)
	Middle-aged (40–64)		0.38	0.32	0.32	0.16
			(0.34–0.43)	(0.25–0.42)	(0.28–0.37)	(0.14–0.17)
Literacy	Illiteracy	Literacy	1.98	2.90	2.08	1.36
			(1.58–2.48)	(2.14–3.95)	(1.68–2.57)	(0.97–1.90)
Spouse	No	Yes	1.04	1.42	1.33	0.66
			(0.92–1.18)	(1.15–1.76)	(1.17–1.52)	(0.62–0.69)
Economic activity	Yes	No	0.82	0.44	0.49	0.77
			(0.74–0.92)	(0.36–0.55)	(0.44–0.56)	(0.73–0.81)
Food-chewing discomfort	Sometimes	Never	2.07	1.76	2.29	1.28
			(1.81–2.36)	(1.37–2.27)	(1.98–2.64)	(1.21–1.36)
	Often		2.56	2.78	3.60	1.62
			(2.22–2.95)	(2.16–3.57)	(3.11–4.16)	(1.50–1.76)
	Always		3.31	4.37	3.92	1.73
			(2.77–3.96)	(3.29–5.82)	(3.26–4.72)	(1.51–1.98)
